# Comparisons of improved genomic predictions generated by different imputation methods for genotyping by sequencing data in livestock populations

**DOI:** 10.1186/s40104-019-0407-9

**Published:** 2020-01-07

**Authors:** Xiao Wang, Guosheng Su, Dan Hao, Mogens Sandø Lund, Haja N. Kadarmideen

**Affiliations:** 1 Quantitative Genomics, Bioinformatics and Computational Biology Group, Department of Applied Mathematics and Computer Science, Technical University of Denmark, Richard Peterson Plads, Building 324, 2800 Kongens Lyngby, Denmark; 2 Center for Quantitative Genetics and Genomics, Department of Molecular Biology and Genetics, Aarhus University, 8830 Tjele, Denmark; 3 Department of Molecular Biology and Genetics, Aarhus University, 8000 Aarhus C, Denmark; 4 College of Animal Science and Technology, Northwest A&F University, Yangling, 712100 Shannxi China

**Keywords:** Genomic prediction, Genotyping by sequencing, Imputation, MAF, Simulation

## Abstract

**Background:**

Genotyping by sequencing (GBS) still has problems with missing genotypes. Imputation is important for using GBS for genomic predictions, especially for low depths, due to the large number of missing genotypes. Minor allele frequency (MAF) is widely used as a marker data editing criteria for genomic predictions. In this study, three imputation methods (Beagle, IMPUTE2 and FImpute software) based on four MAF editing criteria were investigated with regard to imputation accuracy of missing genotypes and accuracy of genomic predictions, based on simulated data of livestock population.

**Results:**

Four MAFs (no MAF limit, MAF ≥ 0.001, MAF ≥ 0.01 and MAF ≥ 0.03) were used for editing marker data before imputation. Beagle, IMPUTE2 and FImpute software were applied to impute the original GBS. Additionally, IMPUTE2 also imputed the expected genotype dosage after genotype correction (GcIM). The reliability of genomic predictions was calculated using GBS and imputed GBS data. The results showed that imputation accuracies were the same for the three imputation methods, except for the data of sequencing read depth (depth) = 2, where FImpute had a slightly lower imputation accuracy than Beagle and IMPUTE2. GcIM was observed to be the best for all of the imputations at depth = 4, 5 and 10, but the worst for depth = 2. For genomic prediction, retaining more SNPs with no MAF limit resulted in higher reliability. As the depth increased to 10, the prediction reliabilities approached those using true genotypes in the GBS loci. Beagle and IMPUTE2 had the largest increases in prediction reliability of 5 percentage points, and FImpute gained 3 percentage points at depth = 2. The best prediction was observed at depth = 4, 5 and 10 using GcIM, but the worst prediction was also observed using GcIM at depth = 2.

**Conclusions:**

The current study showed that imputation accuracies were relatively low for GBS with low depths and high for GBS with high depths. Imputation resulted in larger gains in the reliability of genomic predictions for GBS with lower depths. These results suggest that the application of IMPUTE2, based on a corrected GBS (GcIM) to improve genomic predictions for higher depths, and FImpute software could be a good alternative for routine imputation.

## Background

Genotyping by sequencing (GBS) uses restriction endonucleases to digest genomic DNA and thus sequences digested fragments, and is an efficient method to discover single nucleotide polymorphisms (SNP) [1]. GBS can potentially reduce the cost by producing multiplex libraries [2] and be applied for some species where the commercial chip arrays are not available [3]. Currently, GBS has become a robust genotyping method, but missing genotypes still appear to be a serious problem [1, 4]. Imputation allows the usage of low-density marker panels in the widespread implementation of genomic selection [5]. Thus, imputation strategies are important for using GBS for genomic predictions and many imputation methods have been developed.

Beagle [6] and IMPUTE2 [7] software which are developed for applications in human genetics use a hidden Markov model (HMM) to infer missing markers. The Beagle imputation method constructs a tree of haplotypes and summarizes it in a direct acyclic graph by joining nodes of the tree based on haplotype similarity. The IMPUTE2 imputation method proposes to alternately estimate haplotypes in the reference panel and imputes missing genotypes in the test panel by choosing the most similar estimated haplotypes. FImpute software [8] can achieve accurate imputation when using pedigree information because closer relatives usually share longer haplotypes, while more distant relatives share shorter haplotypes. In a SNP array, missing markers with certain structure are usually identified with a high degree of certainty, but missing markers of GBS data can vary at different marker positions, especially for the mutations located in restriction enzyme cut sites. Therefore, imputation of missing genotypes of GBS SNP data could be less accurate than those in chip SNP data.

As genotype quality influences the reliability of genomic predictions and a low number of reads at a particular marker may create problematic genotypes, genotype data should be edited by discarding problematic data [9] and correcting genotypes of the particular markers with a low number of reads [10]. Minor allele frequency (MAF) is widely used as marker data editing criteria for genomic predictions. Different MAF editing criteria, ranging from 0.01 to 0.05, have been reported to avoid genotyping errors [11–13]. Edriss et al. [9] investigated the effects of editing criteria on the reliability of genomic predictions, using different MAF thresholds. Therefore, it is necessary to investigate the imputation efficiency using different MAF criteria and then confirm the reliability of genomic predictions using imputed data. Although there are many investigations in imputation for chip array data, it is very rare for GBS data. In addition, there is very limited studies on impact of different methods and strategies of imputing missing genotypes (e.g., the approach of imputation followed by genotype correction) in GBS data on genomic prediction.

The objective of this study was to investigate whether genomic predictions (GP) were improved after imputation using three different methods (i.e., Beagle, IMPUTE2 and FImpute software), based on the simulated data of livestock population. Moreover, the accuracies of genomic predictions using different genotype data sets were compared to assess the value of GBS and the improvement from the imputation of missing genotypes.

## Methods

Phenotypic and genomic data of ten replicates for each scenario were generated by QMSim software (version 1.10) [14], using the parameters of population structure (Table 1) and genome (Table 2).

**Table 1:** Simulation parameters of the population structure

Steps	Population structure	Values
	Number of replicates	10
	Overall heritability	0.3
	QTL heritability	0.3
	Phenotypic variance	1.0
Step 1: Historical generations (HGs)	Foundation population size of (HGs)	2000
	Number of generations in phase 1	1000
	Population size in phase 1	2000
	Number of generations in phase 2	200
	Population size in the end of phase 2	400
	The number of males in the last (HG)	200
	The number of females in the last (HG)	200
	Number of males from HG	40
	Number of females from HG	200
Step 2: Expanded generations (EGs)	Number of generations	1
	Litter size	5
	The proportion of male progeny	50%
	Mating design	Random
	Number of males from EG	100
	Number of females from EG	500
Step 3: Recent generations	Number of generations	10
	Litter size	5
	The Proportion of male progeny	50%
	Mating design	Random
	Sire replacement	80%
	Dam replacement	40%
	Selection design	EBV

**Table 2:** Simulation parameters of the genome

Genome	Values
Number of chromosomes	5
Chromosome length	100 cM
Number of marker loci on one chromosome	1,000,000
Marker positions	Evenly
Number of marker alleles in the first HG	2
Marker allele frequencies in the first HG	Random
Number of QTL loci on one chromosome	100
QTL positions	Random
Number of QTL alleles in the first HG	2
QTL allele frequencies in the first HG	Random
QTL allele effect	From a gamma distribution with a shape of 0.4
Marker mutation rate in historical population	2.5 × 10^−5^
QTL mutation rate in historical population	2.5 × 10^−5^

### Population structure and genome

As the domestication process in the historical generations to create linkage disequilibrium (LD), a foundation population of 2000 individuals (1000 males and 1000 females) was maintained at a constant size across 1000 generations and then gradually reduced to 400 individuals over the following 200 generations. Among the 400 individuals in the last generation of the historical population, 40 males and 200 females were randomly chosen for population expansion. In recent generations, 100 males and 500 females from the last generation of the expanded population were selected to continue ten generations. In each generation, a dam reproduced a litter of five individuals. In the whole process of simulation, the individuals of each sex were produced with the equal probability based on the random union of gametes, which were sampled from both the male and female gamete pools. Selection and replacement was performed based on estimated breeding value (EBV), which was estimated by best linear unbiased prediction (BLUP) using animal model [15]. The replacement rate for males and females was 80% and 40%, respectively. The overall heritability, QTL heritability and phenotypic variance were set as 0.3, 0.3 and 1.0, respectively. No remaining polygenic effects were simulated; therefore, all genetic variances were explained by quantitative trait loci (QTLs). The phenotypes were created by adding random residuals to the true breeding values (TBVs), while TBVs were defined as the sum of individual QTL additive effects. The targeted level of LD in this study was close to the values reported for cattle breeds [16, 17] and pigs [18]. The mean r-squared value of LD in the last (10^th^) generation of the recent population was 0.259 (SE = 0.004), based on markers with intervals of less than 50 kb (0 ~ 0.05 cM), averaged over 10 replicates.

Initial LD was created by the process of mutation-drift equilibrium in the historical generations. A total of 5 × 10^6^ SNP markers were evenly distributed on five chromosomes with a size of 100 cM for each. The allele frequency of bi-allelic markers and QTLs was initiated through random sampling from a uniform distribution in the first historical generation. In total, 5 × 100 QTLs were simulated and randomly distributed on these five chromosomes, so 100 QTLs were simulated for each chromosome, which are in the range of most simulation study on genomic prediction. Changing number of chromosomes and number of QTLs could has an influence on prediction accuracy in general, but would not influence the results of comparison between different methods and between scenarios. QTL allele effects were sampled from a gamma distribution with a shape parameter equal to 0.4. The shape parameter used in this study was following Hayes and Goddard [19], as their distribution of estimates of QTL effects were assumed to follow a gamma distribution with shape parameter β = 0.4. In addition, Meuwissen et al. [20] used the same shape parameter to simulate QTL allele effects. To establish mutation-drift equilibriums in historical generations, the marker and QTL recurrent mutation rates in the historical population were both set to 2.5 × 10^− 5^. In the recent populations, no mutation was generated. In general, the simulated population structure and genome were not designed for specific species but the population structure mimicked the population of multiparous species. The results could be useful for most of species, especially the species where the commercial chip arrays are not available such as mink.

### Creating GBS data, quality control and genotype corrections for GBS data

De Donato et al. [21] has reported that the distribution of distances between SNPs differed between chip data and GBS data in cattle. GBS data in cattle showed that 44.0% of SNPs had a distance to the neighboring SNP of less than 50 kb and a distance of more than 150 kb was observed for 13.8% of SNPs. Following De Donato et al. [21], the distribution of fractions (i.e., the distance between neighboring SNPs) in this study were set to 13%, 8%, 8%, 12%, 9%, 6%, 5%, 16%, 7% and 16% for 0.5 kb, 2.5 kb, 7.5 kb, 15 kb, 25 kb, 35 kb, 45 kb, 75 kb, 125 kb and 200 kb, respectively. We generated the called genotype values based on a genotype calling strategy, where a loci was called as homozygous if the reads were for only one allele, and called as heterozygous if the reads were for both alleles. Thus, the called GBS genotypes for homozygous loci were set the same as simulated true genotypes, since all reads presented only one allele under the assumption of no sequencing error. For heterozygous loci, the called GBS genotypes were created according to the number of reads (*n*) and a random number (*r*) from a uniform distribution *r*~*U*(0, 1) [4, 10]. Since the probability of all reads for only one allele (*A* or *a*) was ${\left(\frac{1}{2}\right)}^n$ given true genotype being heterozygote, the heterozygous genotype was replaced by *aa* if $r\le {\left(\frac{1}{2}\right)}^n$, and by *AA* if ${\left(\frac{1}{2}\right)}^n<r<2{\left(\frac{1}{2}\right)}^n$; otherwise, the heterozygous genotype was correctly assigned as *Aa*. Afterwards, GBS loci were set as missing genotypes when reads were equal to zero. An incorrect genotype was expected, where a heterozygous genotype would be wrongly assigned to a homozygous genotype, with a probability of $2{\left(\frac{1}{2}\right)}^n$. In the simulation, read number (*n*) per locus was assumed from a Poisson distribution *n*~*P*(*x*), where *x* was the average depth (*x* = 2, 4, 5, 10).

Quality control criteria included call rates ≥0.8 for individuals and four MAF thresholds for markers before imputation. After quality control, the number of GBS SNPs for no MAF limit, MAF ≥ 0.001, MAF ≥ 0.01 and MAF ≥ 0.03 were approximately equal to 8010 (SD = 6), 7880 (SD = 22), 7540 (SD = 55) and 7100 (SD = 100) averaged over 10 replicates and four depth scenarios, respectively. The difference in number of GBS SNPs among the four depth scenarios were not large. However, the missing genotypes in scenario of average depth = 1 were high, up to approximately 30%; therefore, not many loci met the criteria of call rate ≥ 80%, and this depth was discarded.

The method of genotype correction following the previous method [10] is derived according to Bayes’ formula, *P*(*G*|*GBS*), where *G* is true genotype data (unknown) and *GBS* is GBS data (known), which are subject to genotyping errors. If *GBS*_*aa*_ (*aa* genotype of GBS data) is observed, there are two possible true genotypes (*G*_*aa*_ and *G*_*Aa*_), and the probabilities are

$$ {\displaystyle \begin{array}{l}P\left({G}_{aa}| GB{S}_{aa}\right)=\frac{P\left({G}_{aa}\right)\ P\left( GB{S}_{aa}|{G}_{aa}\right)\ }{P\left( GB{S}_{aa}\right)},\\ {}P\left({G}_{Aa}| GB{S}_{aa}\right)=1-P\left({\mathrm{G}}_{aa}| GB{S}_{aa}\right).\end{array}} $$

Similarly, if *GBS*_*AA*_ was observed, the probabilities are

$$ {\displaystyle \begin{array}{l}P\left({G}_{AA}| GB{S}_{AA}\right)=\frac{P\left({G}_{AA}\right)\ P\left( GB{S}_{AA}|{G}_{AA}\right)\ }{P\left( GB{S}_{AA}\right)},\\ {}P\left({G}_{Aa}| GB{S}_{AA}\right)=1-P\left({G}_{AA}| GB{S}_{AA}\right).\end{array}} $$

If *GBS*_*Aa*_ was observed, then *G*_*Aa*_ is the only possible true genotype, and the probability is

$$ P\left({G}_{Aa}|{GBS}_{Aa}\right)=1. $$

GBS homozygous genotypes were corrected and the resulting genotypes (Gc) were the expected genotype dosage. Let *p* = *P*(*A*) and *q* = *P*(*a*), and 0, 1, and 2 denote genotype *aa*, *Aa* and *AA*. Original GBS genotype are scored as *GBS*_*aa*_ = 0, *GBS*_*Aa*_ = 1 and *GBS*_*AA*_ = 2. Thus,

$$ {\displaystyle \begin{array}{l}G{c}_{aa}=0\times P\left({G}_{aa}| GB{S}_{aa}\right)+1\times P\left({G}_{Aa}| GB{S}_{aa}\right)=\frac{2 pq{\left(\frac{1}{2}\right)}^n}{q^2+2 pq{\left(\frac{1}{2}\right)}^n},\\ {}G{c}_{AA}=1\times P\left({G}_{Aa}| GB{S}_{AA}\right)+2\times P\left({G}_{AA}| GB{S}_{AA}\right)=\frac{2 pq{\left(\frac{1}{2}\right)}^n}{p^2+2 pq{\left(\frac{1}{2}\right)}^n}+2\times \frac{p^2}{p^2+2 pq{\left(\frac{1}{2}\right)}^n}=\frac{2{p}^2+2 pq{\left(\frac{1}{2}\right)}^n}{p^2+2 pq{\left(\frac{1}{2}\right)}^n},\\ {}G{c}_{Aa}=1.\end{array}} $$

Allele frequency can be calculated from the data including all reads. It can also be estimated from GBS genotype data in such a way:

$$ {\displaystyle \begin{array}{l}P\left( GB{S}_{AA}\right)-P\left( GB{S}_{aa}\right)=\left(P\left({G}_{AA}\right)+P\left( GB{S}_{AA}|{G}_{Aa}\right)\right)-\left(P\left({G}_{aa}\right)+P\left( GB{S}_{aa}|{G}_{Aa}\right)\right)={p}^2-{q}^2=2p-1,\\ {}p=\frac{P\left( GB{S}_{AA}\right)-P\left( GB{S}_{aa}\right)+1\ }{2}.\end{array}} $$

### Scenarios of imputation

The imputation was performed in 10,000 individuals across 10 generations using three imputation software: Beagle (version 4.0) [6], IMPUTE2 (version 2.3.2) [7] and FImpute (version 2.2) [8]. IMPUTE2 software allows both the genotype type and genotype dosage as input genotype data. The original GBS genotype type was directly imputed by Beagle, IMPUTE2 and FImpute. Additionally, the expected genotype dosage after genotype corrections was imputed by IMPUTE2 (GcIM). The true genotype of the GBS loci (GBSr) was used for comparison. Finally, genomic predictions were conducted based on GBS data, Beagle imputed data (Be), IMPUTE2 imputed data (IM), FImpute imputed data (FI), GcIM data and GBSr data using four MAF criteria. The missing genotypes for the original GBS were replaced with the mean genotype values for the same loci.

### Accuracy of imputation

Accuracies of imputation were measured using a Pearson correlation and the correct rate of genotype identification. The correlation was defined as the genotypes of GBS data, Beagle imputed data, IMPUTE2 imputed data, FImpute imputed data and GcIM data compared to the true genotype in the GBS loci (GBSr). The correct rate was defined as the non-missing correct genotypes of GBS data, Beagle imputed data, IMPUTE2 imputed data, FImpute imputed data and GcIM data.

### Statistical analysis

Based on the GBS, imputed GBS data and GBSr, genomic estimated breeding values (GEBV) were predicted using the SNP-BLUP model of the BayZ package (http://www.bayz.biz/). The model is

$$ \mathbf{y}=\mathbf{1}\upmu +\mathbf{Mg}+\mathbf{e}, $$

where **y** is the vector of phenotypic values, **1** is the vector of ones, μ is the overall mean, **g** is the vector of random unknown marker effects to be estimated, **M** is the coefficient matrix of genotypes which links **g** to **y**, and **e** is the vector of random residuals. It is assumed that $\mathbf{g}\sim N\ \left(0,\mathbf{I}{\sigma}_g^2\right)$, and $\mathbf{e}\sim N\ \left(0,\mathbf{I}{\sigma}_e^2\right)$.

### Validation

In the 6^th^ to 9^th^ generations of a recent population, 7500 individuals were used as a training set, in which all individuals were genotyped and phenotyped. The test set comprised of 2500 genotyped individuals from the 10^th^ generation. The reliabilities of genomic predictions using marker data from the original GBS, imputed GBS and the true genotypes of GBS loci were compared. The reliabilities of genomic predictions were calculated as squared correlations between the predicted and true breeding values for individuals in the test data set.

## Results

### Missing genotypes and incorrect genotypes

The percentages of missing genotypes at depth = 2, 4, 5 and 10 were 13.5%, 1.83%, 0.673% and 0.00464%, respectively, on average, over ten replicates. The standard deviations (SD) were all less than 4.74 × 10^− 5^. The proportions of incorrect genotypes for depth = 2, 4, 5 and 10 were 15.2%, 7.63%, 4.92% and 0.449%, respectively, and the SDs were all less than 6.32 × 10^− 3^.

### Accuracy of imputation based on original GBS data

**Fig. 1:**
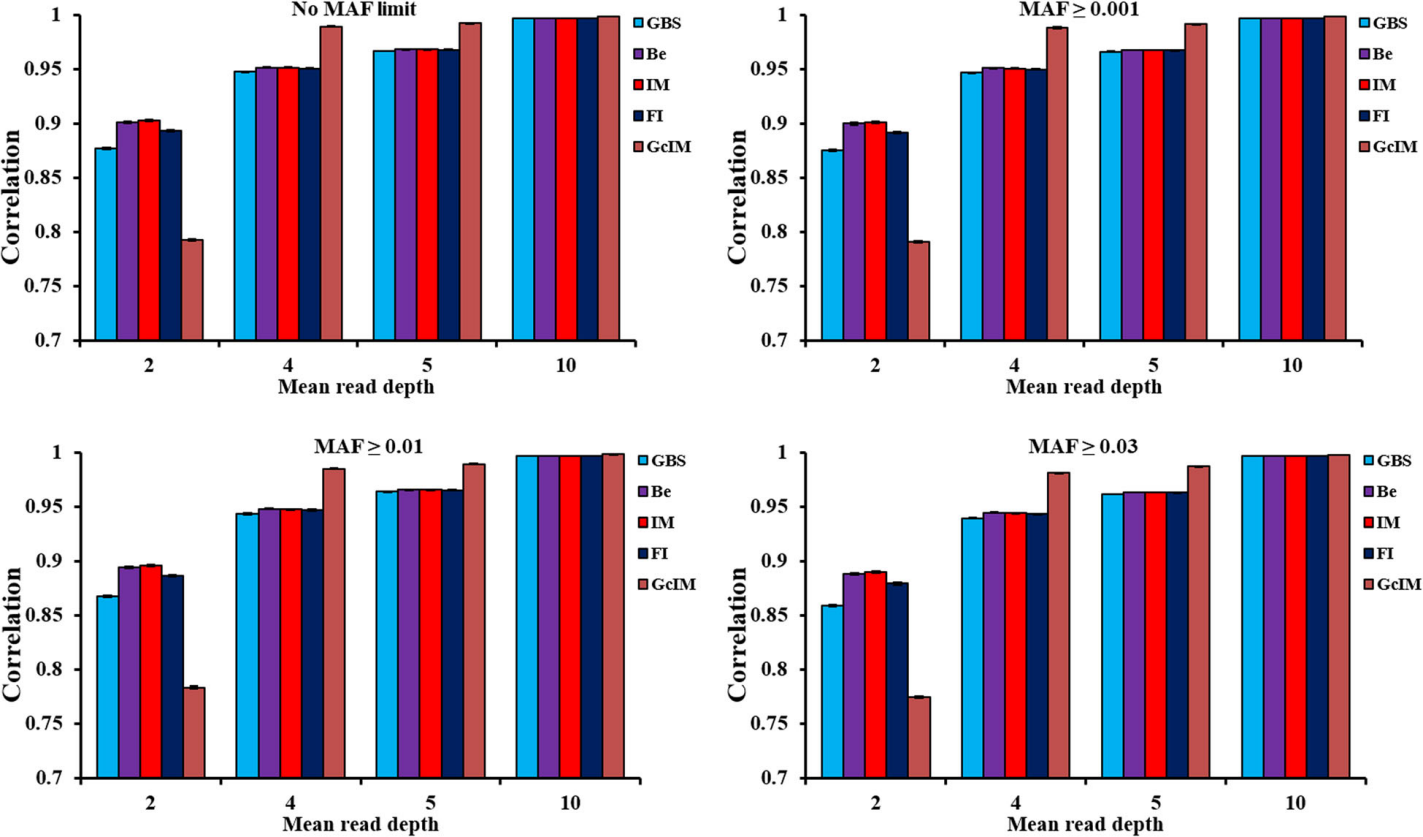
Correlations for the original GBS (GBS), the Beagle imputed genotypes (Be), the IMPUTE2 imputed genotypes (IM), the FImpute imputed genotypes (FI) and the imputed genotypes based on corrected GBS (GcIM). Note: MAF criteria were used to delete markers with low MAF values before imputation

As shown in Fig. 1, there were very small differences in the imputation accuracy among the four GBS data sets based on the MAF criteria used (no MAF limit, MAF ≥ 0.001, MAF ≥ 0.01 and MAF ≥ 0.03). In addition, imputation accuracy was higher for larger depth, regardless of the imputation method used. For MAF ≥ 0.01, the imputation accuracies for depth = 2, 4, 5 and 10 were 0.868, 0.943, 0.964 and 0.997, respectively, using GBS data without imputation; 0.894, 0.948, 0.965 and 0.997, respectively, using Beagle; 0.896, 0.947, 0.965 and 0.997, respectively, using IMPUTE2; and 0.886, 0.947, 0.965 and 0.997, respectively, using FImpute. The imputation accuracies were the same for the three imputation methods, except for when using the data of depth = 2, where FImpute had a slightly lower imputation accuracy than the other methods. However, FImpute only took several minutes for each imputation procedure, while Beagle took 12–14 h and IMPUTE2 took 12–41 h, depending on the depth. Overall, the imputation time decreased with the depth increased, due to fewer missing genotypes.

### Accuracy of imputation based on corrected GBS data

**Fig. 2:**
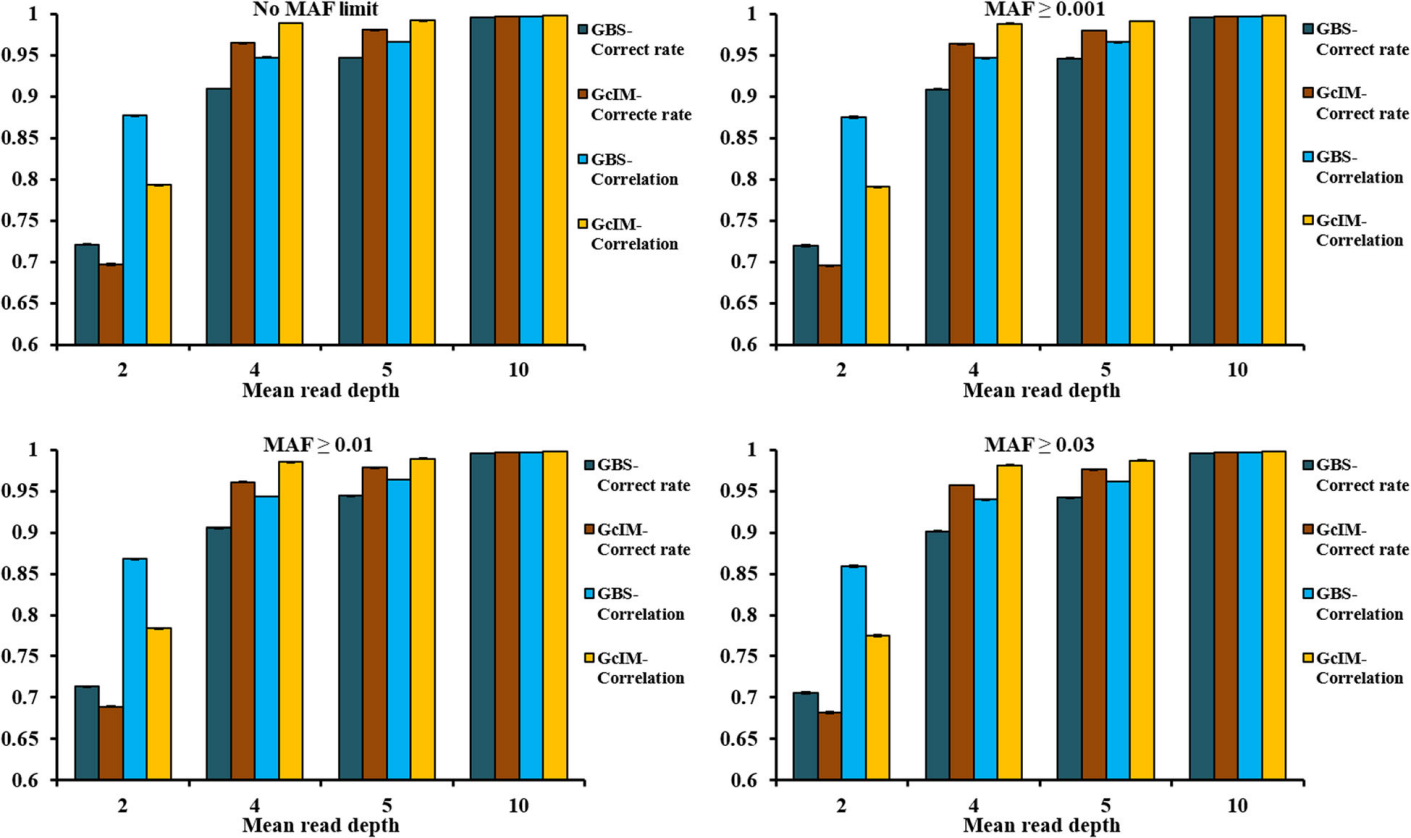
Correct genotype rates and correlations for GBS and imputed genotypes based on corrected GBS (GcIM). Note: MAF criteria were used to delete markers with low MAF values before imputation

The imputations based on corrected GBS (GcIM) data were observed to be the best for all imputation methods at depth = 4, 5 and 10 but were the worst at depth = 2 (Fig. 1). For MAF ≥ 0.01, the corrected genotype rates for depth = 2, 4, 5 and 10 were 0.713, 0.905, 0.944 and 0.995, respectively, using GBS data without imputation; and 0.689, 0.961, 0.978 and 0.997, respectively, using GcIM (Fig. 2). Correlations were 0.868, 0.943, 0.964 and 0.997, using GBS data without imputation; and were 0.784, 0.985, 0.989 and 0.998, using GcIM, for depth = 2, 4, 5 and 10, respectively (Figs. 1, and 2). Obviously, both the corrected genotype rates and the GcIM correlations showed lower values than GBS at depth = 2.

### Reliability of genomic prediction

**Fig. 3:**
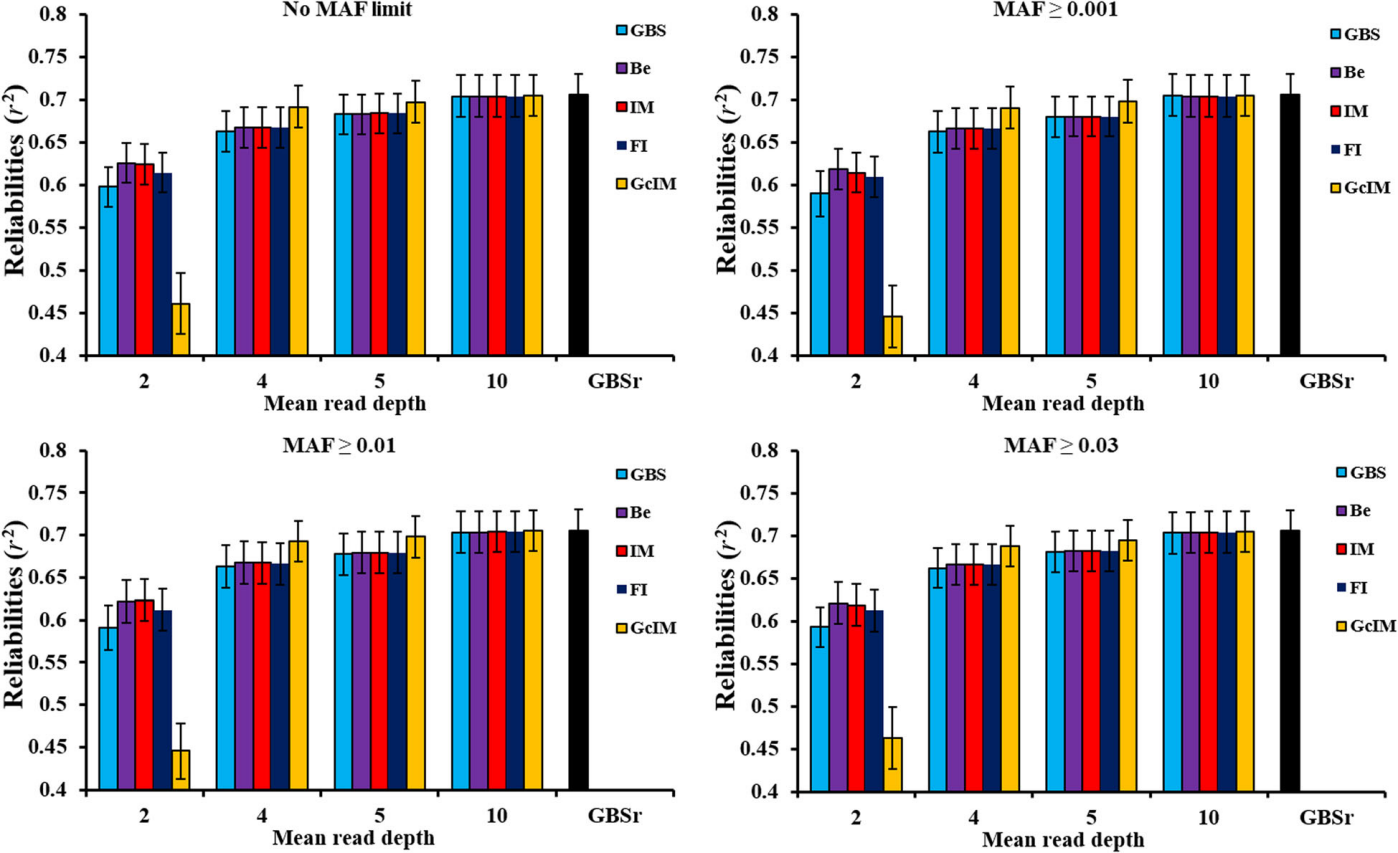
Reliabilities (*r*^2^) of genomic predictions using original GBS (GBS), GBS with imputation of missing genotypes (Be, IM, FI), imputed corrected genotype by IMPUTE2 (GcIM) and true genotypes of GBS markers (GBSr), at four depths, averaged over 10 replicates. Bars indicate SE. Note: MAF criteria were used to delete markers with low MAF values before imputation

**Fig. 4:**
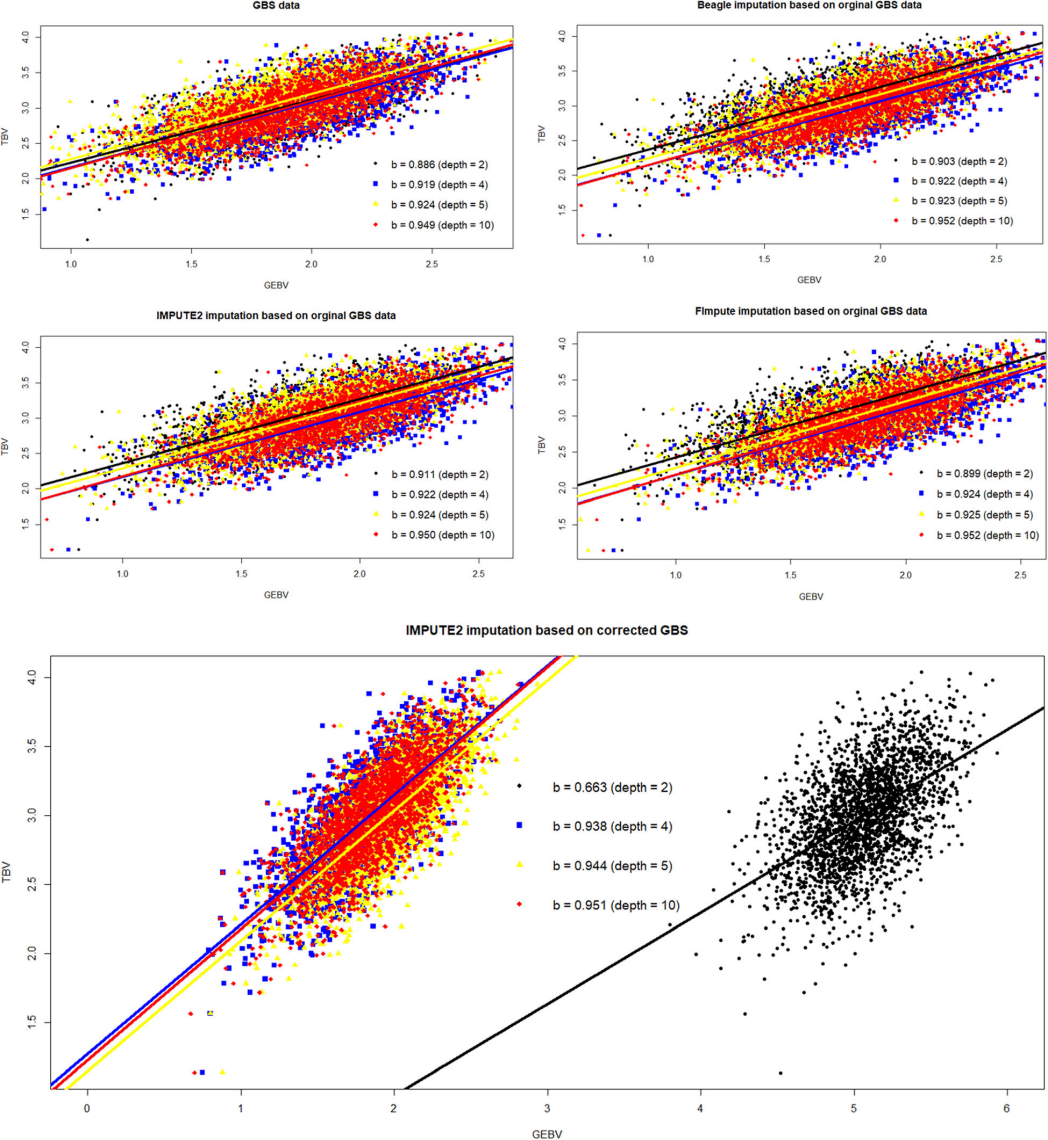
Regression of true breeding value (TBV) on genomic estimated breeding values (GEBV). Note: b is the regression coefficient

The reliabilities (*r*^2^) of genomic predictions using GBS data, without imputation, at depth = 2 were 0.598, 0.590, 0.591 and 0.593 for no MAF limit, MAF ≥ 0.001, MAF ≥ 0.01 and MAF ≥ 0.03, respectively. Thus, almost no differences existed among the four criteria for quality control, and retaining more SNPs could result in higher prediction reliability (Fig. 3). The prediction reliabilities using true genotypes in the GBS loci (GBSr) for the four MAF criteria were all 0.706. As depth increased from 2 to 10, the prediction reliabilities using GBS and imputed genotypes, following the three imputation methods, gradually approached the reliabilities using true genotypes (GBSr). Imputation improved genomic prediction to different degrees, consistent with the accuracy of imputing missing genotypes (Fig. 1). Compared with GBS data without imputation, Beagle and IMPUTE2 resulted in an increase of the prediction reliability of 5 percentage points, while FImpute gained 3 percentage points, at depth = 2 (Fig. 3). The trend in the reliabilities of genomic predictions using GcIM were consistent with its genotype accuracy (correlations and correct rates) after imputation at four depths (Fig. 2). Among the five sets of GBS data, GcIM led to best prediction at scenarios of depth = 4, 5 and 10, but worst at depth = 2. The reliability of genomic prediction using GcIM were 0.693, 0.698 and 0.705 for depth = 4, 5 and 10, respectively, using MAF ≥ 0.01, approaching the reliabilities using true genotypes (GBSr). The standard error (SE) of the prediction reliabilities in the 10 replicates was approximately 0.025. Figure 4 showed the regression of true breeding values (TBV) on genomic estimated breeding values (GEBV). With the same trend of prediction reliabilities, regression coefficients increased as the depth increased from 2 to 10. Meanwhile, Beagle, IMPUTE2 and FImpute resulted in higher regression coefficients. The lowest regression coefficient far from one (0.663) was also found using GcIM at depth = 2 (Fig. 4).

The FImpute method is able to use pedigree information, so we compared FImpute imputation with pedigree and without pedigree information for scenario of depth = 4. In this analysis, genotypes were corrected, and after correction, the expected genotype value were rounded to integral genotype code. Then, the missing genotype were imputed using FImpute with or without pedigree information (GcFIped or GcFI). It showed that imputation with pedigree information led to a slightly higher prediction reliability, compared with imputation without pedigree (Table 3). On the other hand, imputation did not improve prediction accuracy when kept all genotypes without considering number of reads. However, when setting the genotypes with read = 1 & 2 as missing and then performing imputation, the prediction reliabilities of this scenario was two percentage points higher than that keeping genotypes with read = 1 & 2 (Table 3).

**Table 3:** Imputation accuracies compared to true genotypes in the GBS loci (GBSr) and reliabilities of genomic prediction using GBS data imputed by FImpute with or without pedigree information (GcFIped or GcFI) at depth = 4 and MAF ≥ 0.01, averaged over 10 replicates. The imputation was performed after genotype correction (i.e., GcFIped and GcFI). Note: Depth = 4 (1) or Depth = 4 (1 & 2) indicated the genotypes with read = 1 or 1 and 2 were set as missing genotypes. Standard errors were shown within bracket

	Depth = 4		Depth = 4		Depth = 4 (1)		Depth = 4 (1 & 2)	
	FI	FIped	GcFI	GcFIped	GcFI	GcFIped	GcFI	GcFIped
Correct genotype rate	0.905 (< 0.0005)	0.920 (< 0.0005)	0.915 (< 0.0005)	0.919 (< 0.0005)	0.927 (< 0.0005)	0.935 (< 0.0005)	0.942 (< 0.0005)	0.950 (< 0.0005)
Correlation	0.946 (< 0.0005)	0.955 (< 0.0005)	0.951 (< 0.0005)	0.954 (< 0.0005)	0.960 (< 0.0005)	0.965 (< 0.0005)	0.971 (< 0.0005)	0.976 (< 0.0005)
Prediction reliability	0.666 (0.0253)	0.670 (0.0245)	0.666 (0.0248)	0.668 (0.0245)	0.674 (0.0246)	0.679 (0.0246)	0.683 (0.0242)	0.689 (0.0246)

## Discussion

### Impact of editing marker data using different MAF criteria on genomic predictions

As SNP marker effects with low MAF values cannot be estimated accurately, they are usually eliminated to improve the estimating stability of the remaining SNP effects during genomic predictions [22]. In the previous studies, the MAF was usually used as a criterion to edit marker data for genomic predictions with thresholds ranging between no limit and 0.1 [9, 11-13, 23]. In this study, the numbers of SNPs in the edited data before imputation were approximately equal to 8010, 7880, 7540 and 7100 using the four MAF criteria of no MAF limit, MAF ≥ 0.001, MAF ≥ 0.01 and MAF ≥ 0.03, respectively. These settings led to reductions in the number of SNPs but resulted in small differences in the accuracies of genomic predictions. Generally, it appeared that retaining more SNPs resulted in higher prediction reliabilities (Fig. 3). This is consistent with other research showing that an increasing MAF threshold led to a reduction in prediction reliability [9], suggesting that SNP markers with low MAF values do not harm genomic prediction.

### Imputation of missing genotypes based on original GBS data

Imputation based on original GBS showed that correlations increased with increasing GBS depth (Fig. 1). These three imputation methods made no changes for non-missing markers; therefore, the rates of genotype recognition were as same as GBS. As a whole, Beagle and IMPUTE2 performed better than FImpute at depth = 2 (Fig. 1). In the previous studies, it was reported that Beagle performed best for imputations from 5 K to 50 K in Angus cattle [23] and from 3 K to 54 K in a mixture of two Red cattle populations [24]. However, when the relationship between individuals was stronger and the number of genotyped animals was larger, FImpute outperformed Beagle for imputations from 3 K to 54 K in a combined cattle population [25]. A large distance between GBS SNP markers in some regions with weaker LD might affect imputation accuracy because LD is an important information factor used by imputation methods to infer unobserved genotypes [26]. When using IMPUTE2 software for imputation in this study, five chromosomes were imputed separately instead of splitting a chromosome into several segments. Splitting could cause LD information between segments not to be used for imputation [23], which could be the reason that IMPUTE2 performed better than FImpute in this study. The results were also consistent with the research of Ma et al. [24] that did not split a chromosome and the other studies that split a chromosome into several segments [26, 27]. However, due to high computational demands, splitting into segments is usually considered. For practical use, a larger number of animals and the whole genome will be genotyped, so imputation time could be very important for the whole process. FImpute software could perform as well as Beagle and IMPUTE2 software for the imputation of GBS data with a depth larger than 2 while consuming much less time. Imputation time was mostly influenced by the sample size [28] and the percentage of missing genotypes, dependent on depth. Generally, Beagle and IMPUTE2 software could take more than 12 h for each imputation, while FImpute software required just several minutes in this study. In other studies, FImpute also performed in the range of minutes, while Beagle and IMPUTE2 took hours or longer [24, 29].

### Imputation of missing genotypes based on corrected GBS data

Posterior probabilities after corrections of genotypes (Gc) could be more close to the true genotypes, likely because rounding posterior probabilities into integral types could cause the loss of meaningful information regarding uncertainty [30]. Imputation is a prediction process; therefore, incorporating quantified uncertainty can increase imputation accuracy [31]. The IMPUTE2 imputation based on corrected GBS (GcIM) was observed to perform the best out of all the imputation methods based on the original GBS at depth = 4, 5 and 10. Thus, the imputation of GcIM had the correct genotype rate of 0.961, 0.978 and 0.997, and the correlations (between imputed and true genotypes) of 0.985, 0.989 and 0.998 in scenario at depth = 4, 5, and 10, respectively, for the MAF ≥ 0.01 (Fig. 2). However, GcIM also led to an unexpectedly worse imputation at depth = 2, and thus, the correct genotype rate was 0.689 and the correlation was 0.784 in the MAF ≥ 0.01 scenario at depth = 2 (Fig. 2). The most frequent haplotype will usually be imputed when a haplotype cannot be determined clearly; therefore, it was suggested that closer relatives shared incorrect longer haplotypes as frequent haplotypes for GBS data of low depths, which caused unexpected imputation results [32].

### Impact of imputation on genomic predictions

Imputation could recover the loss of information in low-coverage GBS data and increase the reliability of genomic predictions [33, 34]. Previous studies showed that prediction reliabilities using imputed genotypes were slightly lower than those using true genotypes [35, 36], which is consistent with our genomic prediction results at depth = 4, 5 and 10. Based on the original GBS in this study, Beagle and IMPUTE2 resulted in an increase in the imputation correlation by 3 percentage points, and FImpute gained 2 percentage points at depth = 2 (Fig. 1). The consistent reliabilities of genomic predictions increased 5 percentage points after Beagle and IMPUTE2 imputation and 3 percentage points after FImpute imputation (Fig. 3). The previous study showed that the reliabilities of genomic prediction using corrected GBS were 0.604, 0.672, 0.684 and 0.704, with the improved values of 0.013, 0.009, 0.006 and 0.001 after genomic correction at depth = 2, 4, 5 and 10, respectively [10]. IMPUTE2 imputation based on corrected GBS (GcIM) increased by 0.056, 0.034 and 0.002 for correct genotype rates, and 0.042, 0.025 and 0.002 for correlations at depth = 4, 5 and 10, respectively, in the MAF ≥ 0.01 scenario (Fig. 2). The consistent reliabilities of genomic predictions for GcIM at depth = 4, 5, and 10 also increased by 0.03, 0.02 and 0.002, respectively. In total, the level of improvement in the reliability of genomic prediction was consistent with the level of improvement in the accuracy of imputation. Compared to the original GBS in this study, the accuracy of imputation based on the corrected GBS decreased 0.024 for correct genotype rates and 0.084 for correlations at depth = 2 for the MAF ≥ 0.01 scenario (Fig. 2). This could explain the decline in the reliability of genomic prediction from 0.591 to 0.446 (Fig. 3) and in the regression coefficient from 0.886 to 0.663 (Fig. 4). This result suggests that IMPUTE2 imputation based on corrected GBS performed best and increased the reliability of genomic predictions most at higher depths but not at lower depths. Except for GcIM, imputation resulted in very tiny or no increase in quality of SNP data and reliability of genomic prediction when average depth was four or more. The possible reason could be that the poor genotypes stayed in the dataset without being removed away, such as the genotypes with one or two reads. In Table 3 of this study, the prediction reliabilities increased when setting the genotypes with read = 1 & 2 as missing in scenario of depth = 4. The results suggest that it is a good strategy to set genotypes with low number of reads (e.g., ≤2) as missing genotypes and then impute the missing genotypes before genomic prediction.

Our study investigated the imputation of missing genotypes for the individuals with the same designed depth of GBS data, which is important for both genome-wide association study (GWAS) and genomic prediction, especially for using low depth of GBS data. In commercial applications, combinations of high-density and low-density SNP arrays have been used to reduce the genotyping costs. Similarly, GBS data may include individuals genotyped with different depths. Therefore, it is also interesting to investigate the impact of imputation when subset of the individuals have low read depths and the other subset of the individuals have high read depths. It is expected that the joint imputation will increase the accuracy of imputation and prediction for the individuals with low read depths. However, the hypothesis needs further studies.

## Conclusions

The current study compared imputation methods for GBS genotypes and improvements in genomic predictions from the imputation of missing markers. The results showed that imputation accuracy was relatively low for GBS at a low depth (approximately 0.90 for depth = 2) and high for GBS at a high depth (approximately 0.96 for depth = 5). In addition, imputation resulted in larger gains in the reliability of genomic predictions for GBS at a low depth, which had a larger number of missing genotypes. These results suggest application of GcIM to improve genomic predictions at higher or intermediate depths. In addition, FImpute software could be a good alternative for practical and routine imputation because of low computational demands.

## Data Availability

The datasets used in the current study are available from the first author Xiao Wang: xiwa@dtu.dk and the corresponding author Haja N. Kadarmideen: hajak@dtu.dk

## Abbreviations

Be: Beagle imputation based on original GBS
cM: Centimorgan
Depth: Sequencing read depth
EBV: Estimated breeding value
EG: Expanded generation
FI: FImpute imputation based on original GBS
GBS: Genotyping by sequencing
GBSr: True genotype in the GBS loci
Gc: Correction of genotype
GcIM: IMPUTE2 imputation based on corrected GBS
GEBV: Genomic estimated breeding value
GP: Genomic prediction
GWAS: Genome-wide association study
HG: Historical generation
HMM: Hidden Markov model
IM: IMPUTE2 imputation based on original GBS
LD: Linkage disequilibrium
MAF: Minor allele frequency
QTL: Quantitative trait loci
SD: Standard deviation
SE: Standard error
TBV: True breeding value
